# Developing retinal biomarkers of neurological disease: an analytical perspective

**DOI:** 10.2217/bmm.15.17

**Published:** 2015

**Authors:** Ian JC MacCormick, Gabriela Czanner, Brian Faragher

**Affiliations:** 1 Department of Eye & Vision Science, University of Liverpool, Liverpool, UK; 2 Malawi-Liverpool-Wellcome Trust Clinical Research Programme, Blantyre, Malawi; 3 Centre for Clinical Brain Sciences, University of Edinburgh, Edinburgh, UK; 4 Department of Biostatistics, University of Liverpool, Liverpool, United Kingdom; 5 Liverpool School of Tropical Medicine, Liverpool, UK

**Keywords:** biomarker, brain, cerebral malaria, proxy marker, retina, surrogate end point

## Abstract

The inaccessibility of the brain poses a problem for neuroscience. Scientists have traditionally responded by developing biomarkers for brain physiology and disease. The retina is an attractive source of biomarkers since it shares many features with the brain. Some even describe the retina as a ‘window’ to the brain, implying that retinal signs are analogous to brain disease features. However, new analytical methods are needed to show whether or not retinal signs really are equivalent to brain abnormalities, since this requires greater evidence than direct associations between retina and brain. We, therefore propose a new way to think about, and test, how clearly one might see the brain through the retinal window, using cerebral malaria as a case study.

## Background

### The appeal of the retina as a research tool

There is a great temptation to describe the retina as a ‘window to the brain’. For example, a recent review of neurological conditions was titled ‘The retina as a window to the brain – from eye research to CNS disorders’ [1]. A popular textbook on retinal anatomy and physiology is called: ‘The retina – an approachable part of the brain’ [2]. Similar language and concepts are present in recent literature on Alzheimer’s disease [3], schizophrenia [4] and stroke [5].

The appeal of the retina as a neuroscientific research tool arises from three points: first, it is difficult to directly observe the brain in living patients. This limits the amount and type of information that can be collected about the CNS in health and disease. Second, the retina is thought to be similar to the brain [6,7]. Although important differences exist (e.g., photoreceptor and neuron metabolism) [2], the retina is part of the central nervous system (CNS) and has similar embryological origins, anatomy and physiology to other CNS regions. This leads to a related idea that retinal disease manifestations ought to be associated with brain disease manifestations – at least for certain conditions, where both organs are exposed to the same insults. Finally, unlike the brain, direct observation of the retina is relatively simple. Several noninvasive high resolution techniques to measure retinal structure and/or function are available [8], and technological advances are likely to increase the range and power of such modalities.

Given these points, it is not surprising that the retina has been the subject of a significant amount of biomarker research, leading to many reports of associations between retina and brain. Prominent associations include relationships between retinal parameters and outcomes from several neurological conditions, including stroke [9], cognitive impairment [3,10], multiple sclerosis [11] and others (reviewed in [1,8]).

It is clear that associations between retina and brain exist for a range of neurological and neurovascular conditions of varying etiologies. This is consistent with the hypothesis that the retina and brain are similar, and respond similarly to disease. However, although evidence of consistency with a hypothesis is valuable, associations between retina and brain cannot – on their own – justify the stronger conclusion that retinal markers mirror analogous brain features, or, in some sense, provide a window to the brain.

The reason for this has to do with what it would mean for the retina to be a window to the brain.

### The retina as a window to the brain Reasoning from an analogy between retina & brain

The phrase ‘window to the brain’ suggests that some characteristic in the retina, or retinal manifestation of disease, is the same as an analogous characteristic or manifestation in the brain. It suggests that when one observes a retinal feature, one is also observing, for all practical purposes, an equivalent or identical disease effect that is occurring in the brain. If this was true, we should certainly expect the given retinal and brain variables to be strongly positively correlated. But proof of equivalence requires evidence beyond direct association.

Consider two variables, S and T. If S and T are materially equivalent (symbolized S⇔T), then S is necessary and sufficient for T, and *vice versa*. If the retina is essentially a transparent ‘window to the brain’, this type of relationship ought to exist between retinal and brain variables. To the best of our knowledge, existing reports of retinal biomarkers of brain disease usually describe univariate or multivariate associations between retinal and brain variables, but this does not show that a retinal marker is necessary and sufficient for an analogous brain feature.

The point to take from this is not that authors describing the retina as a window are wrong – direct associations between retina and brain are important. On the contrary, this is an opportunity. Considering the many biologically important characteristics shared by retina and brain, we ought to be able to describe retina–brain relationships in much stronger terms than those of simple associations.

What would this kind of description look like? The strict logic of material equivalence may not be appropriate for analysis of a biological system. Even if a retinal variable really was necessary and sufficient for an analogous brain variable, there are many reasons why this relationship might not be demonstrable. These include measurement error of key variables, and complexity introduced by other related factors such as confounders. Biology is a noisy science.

Define equivalence (S⇔T) to mean: S contains the same information as T. The information contained in S (and also T) is necessarily relative to information outside itself. About what does S inform us? We are interested in retinal biomarkers because they might inform us about other variables. These might include:

- A systemic disease process (A);
- Analogous manifestation(s) of this disease process in the brain (T);
- The ultimate outcome of the disease process (e.g., risk of death) (Z).

If S⇔T means S contains the same information as T, then this can be expressed as:

- S and T contain the same information about each other and, in addition,
- S and T both contain the same information about A and Z.

However, since biology is noisy, we might want to define equivalence in even more pragmatic terms. Instead of defining equivalence on the basis of whether or not S and T contain alI the same information about each other, A and Z, we can define it in terms of the degree to which S informs about T, with respect to A and Z.

**Figure 1:**
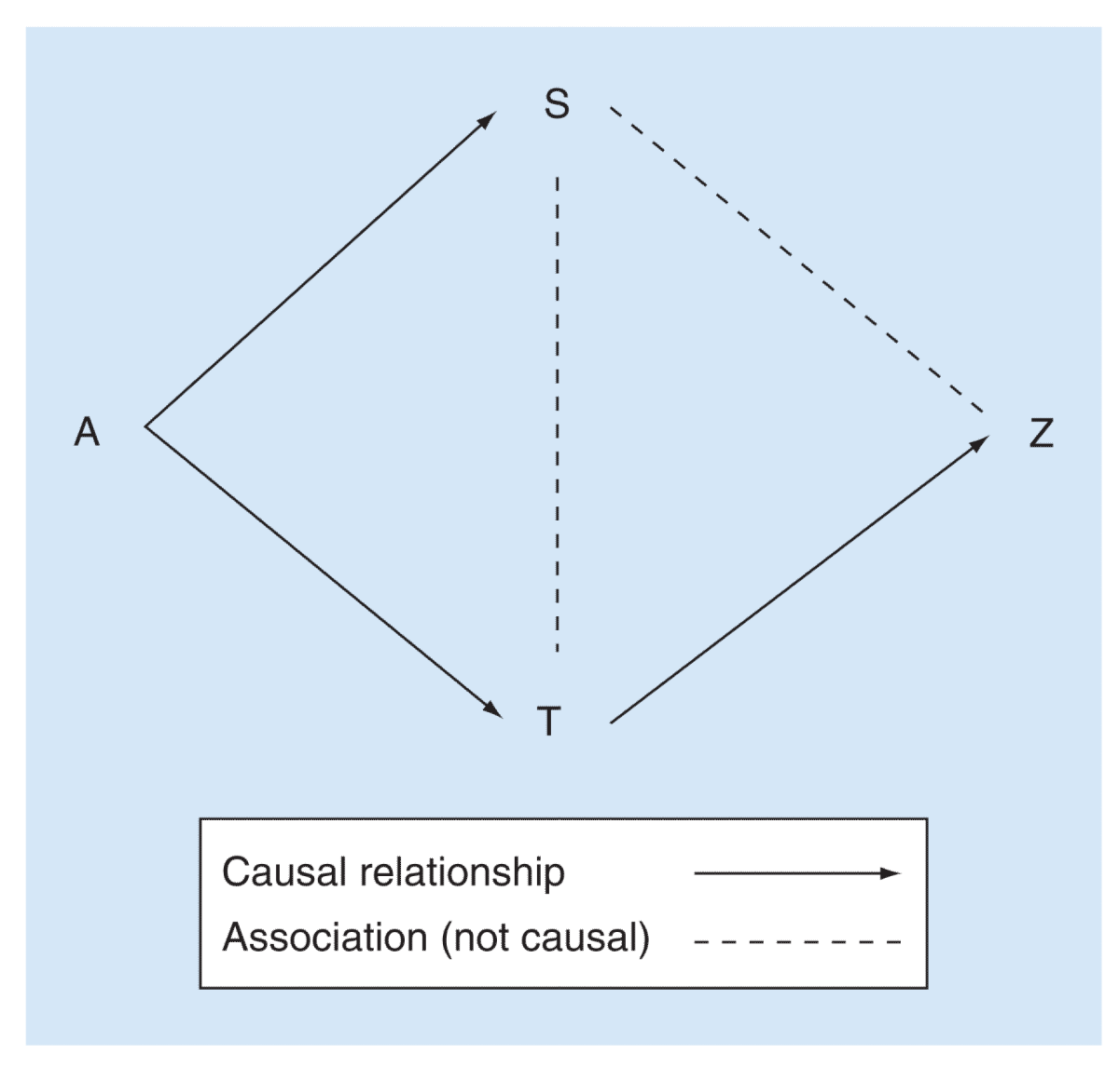
Modified directed acyclic graph illustrating how equivalence between retina and brain variables can be thought of in terms of information contained in the retina (S) about the brain (T) with respect to disease exposures and effects (A and Z, respectively). Arrows indicate hypothesized causal relationships; broken lines indicate direct associations that are assumed not to be causal. A: disease exposure; S: source domain variable (e.g., a retinal variable); T: target domain variable (e.g., a brain variable); Z: clinical outcomes. If a retinal feature (S) is analogous to a brain feature, it ought to contain a large amount of information about the brain feature (T) both independently and with respect to the mechanism causing disease manifestations (A) and the clinical outcome (Z). This figure also illustrates a biological paradigm relating disease exposure (A) to disease manifestations in retina (S) and brain (T), and finally to clinical outcome (Z) for pediatric cerebral malaria. Note, there is no direct path from A to Z. This implies that in cerebral malaria, the disease (A) only causes death (Z) through manifestations in the brain (T). The paradigm can be modified to include different assumptions, and these pathways can be represented mathematically as a series of simultaneous equations in a structural equation model. S can be evaluated in terms of how much information it contains about A or Z, compared with T (cf. LRF, or PIG). It can also be evaluated in terms of the ratio of coefficients: A → S / A → T, or S – Z / T → Z (cf. relative effect).

In this case, a retinal feature could be considered equivalent to an analogous brain feature, if the retinal feature contained a large amount of the information about disease exposure (A), and/or disease outcome (Z), as that contained in an analogous brain feature (T). The retinal feature (S) and brain feature (T) should also contain a large amount of information about each other. These relationships are illustrated in Figure 1.

These considerations of what it might mean for the retina to be a window to the brain lead to a definition of equivalence that moves some way beyond existing analyses of prospective retinal biomarkers in terms of simple associations with brain variables or outcomes. However, they are not unique to questions about retina–brain associations.

Similar analogical reasoning is used when assessing the suitability of animal models, and in evaluating potential surrogate markers in randomized controlled trials (RCTs). In each of these examples, the real object of interest is out of reach. This is the target (T) – for example, the human subject, or the true clinical end point. What is available is an imperfect source of information (S) – a candidate model, or prospective surrogate marker. S may (or may not) provide useful information about the real object of interest (T). In both cases, the biological plausibility of relationships between S, T, A and Z gives an essential *a priori* context to any empirical evaluation of S as a good source of information about T – whether S is an experimental model, surrogate marker or ‘window’ to another organ.

Recognizing that analogical reasoning is used in other areas of medical science suggests the possibility that existing statistical methods might be adapted for use in observational studies of retinal variables as potential biomarkers for brain disease. Taking statistical techniques developed to assess surrogacy in RCTs, and extending them to observational studies, could potentially allow important new inferences to be made about the pathogenesis and prognosis of neurological diseases from retinal data. Indeed, this methodology could be applied much more broadly. For example, a recent study used similar methods to simultaneously estimate the effect of bacteria strain on both biomarkers and mortality [12].

However, questions about whether or not retinal observations reflect cerebral disease processes are different from questions about treatment effect in an RCT. Direct application of statistical methods from RCTs to observational studies are, in general terms, not usually appropriate. Analytical approaches that allow fuller exploration of potentially causal relationships are more suitable – for example, structural equation modeling (SEM) methods. SEM has the advantage of allowing researchers to declare assumptions about relationships between exposures, outcomes, and potential confounders, and take account of these relationships when estimating coefficients (for more details, see [13,14]).

Even if one or more statistical methods for evaluating surrogacy in RCTs are appropriate for use in observational studies, certain theoretical misconceptions about surrogacy must be avoided. For example, Walker *et al.* [12] discuss how, because their methodology is conceptually similar to assessment of surrogate end points, they can conclude that the associations they describe represent causal relationships. This is incorrect. A surrogate marker may, or may not be on the causal pathway between treatment (or disease) and true end point. Mediation is not a necessary condition for surrogacy [15,16].

This comment may seem esoteric, but it has very practical implications when considering the retina as a model of the brain. It does not make biological sense to think of the retina mediating the effect of a systemic process on the brain. If mediation is logically necessary for surrogacy, retinal manifestations might be better thought of as epiphenomena than glimpses of the brain through the supposed window of the eye.

A further caution about surrogate evaluation is relevant. Surrogate end points are attractive because they are easier, quicker or cheaper to measure than the true clinical end point, while providing effectively the same information. New medicines can potentially reach the market more quickly and cheaply if licensing is based on surrogate outcomes rather than true clinical end points such as mortality. This was true of early antiretroviral drugs, where CD4^+^ count was used as a surrogate for development of AIDS (discussed in [17]). On the other hand, there is also a serious danger of being misled by a surrogate, because apparently good surrogate end points can produce paradoxical results. This occurred to disastrous effect when antiarrhythmic drugs were licensed because they suppressed arrhythmia, but were later found to increase mortality (discussed in [18,19]). If this type of paradox can occur in the context of RCTs, it can certainly cause problems for interpreting the meaning of retinal markers of brain disease.

## Evaluation of analogical reasoning: surrogate end points

Evaluation of surrogate end points is an active area of statistical research, and several definitions and operational criteria have been proposed (reviewed in [15–17,20,21]). We summarize some of the more prominent approaches, with the aim of identifying statistical methods to investigate relationships between retina and brain within the context of a particular disease exposure and outcome (Table 1).

## Prentice’s definition & criteria

Prentice [22] suggested a highly influential definition for surrogate end points, with operational criteria by which a variable could be tested statistically (Table 1). This became a fundamental reference point for many, if not all, subsequent authors on statistical evaluation of surrogate end points. His definition was based on the intuitive concepts that a good surrogate should:

- Be associated with the true end point, and also,
- ‘Capture treatment differences as they affect the true end point’ ([22], page 433).

Prentice’s definition and criteria formalized an important intuition about what it means for a surrogate outcome to be valid and were foundational to later discussions about how to evaluate surrogate end points. However they suffer from several major problems. The main requirement is that the surrogate acts as a perfect confounder for the relationship between treatment and true outcome. This is a very high, and probably unrealistic standard. Formulation in terms of hypothesis testing means that although the criteria may be suitable for invalidating poor surrogates (given adequate power), they can never truly validate a good surrogate, since this requires the null hypothesis to be proved true instead of false [23]. The criteria are only equivalent to Prentice’s definition of validity for binary end points [24]. They do not allow for confounders of the relationship between surrogate and true outcome, and do not exclude the type of surrogate paradox involved in the infamous arrhythmia trials [16].

## Proportion explained

Several adaptations of Prentice’s criteria have been proposed. Freedman *et al.* (1992) [23] developed the ‘proportion explained’ (PE) (Table 1). This uses Prentice’s definition of a valid surrogate, but instead of testing whether or not the surrogate captures the entire effect of treatment on the true end point, the statistic measures the degree to which a surrogate end point captures treatment effect. It was designed as a ratio of treatment effect on the true outcome, given the surrogate, to unadjusted treatment effect on the true outcome.

This addresses one of the main criticisms of Prentice’s criteria. Unfortunately, this statistic also suffers from several major problems. It is not a true ratio – it is larger than one if control for the surrogate changes the direction of treatment effect on the true outcome. Confidence intervals will be large unless the treatment effect on the true outcome is also very large (>4 standard errors). These issues make interpretation of the PE difficult [24]. Furthermore, a close association between the treatment and surrogate leads to large variability in the numerator. Although one might expect a good surrogate outcome to be closely associated with the treatment, the PE functions best when there is no interaction between treatment and surrogate [20,25].

## Likelihood reduction factor & proportion of information gain

Another approach to Prentice’s criteria is the ‘likelihood reduction factor’ (LRF) [26] (Table 1). This was proposed within the context of a different concept of surrogacy, often referred to as the ‘meta-analytic approach’ (discussed below) [16]. Alonso *et al.* [26] suggested that the LRF could bridge these two concepts – on one hand, providing a statistic to evaluate Prentice’s main criterion (treatment effect on the true outcome is captured by the surrogate), and on the other, an expression of ‘individual level surrogacy’ that could be applied to a range of data types from a single trial.

Like the PE, the LRF compares two models: a model of treatment effect on the true outcome (controlling for the surrogate), and an unadjusted model of treatment effect on the true outcome. The PE compares coefficients of the models; the LRF compares the log likelihoods of fitted models. In some families of model (e.g., logistic), the LRF can only take a value between zero and a number less than one. An adjusted version of the LRF (LRF_adj_) will always lie between zero and one. Unlike the PE and LRF, the LRF_adj_ appears to be relatively unaffected by interaction between treatment and surrogate [25].

Qu & Case (2007) [25] proposed a variation of the LRF_adj_. This statistic, the ‘proportion of information gain’ (PIG), compares models using the likelihood ratio test (Table 1). Results from these tests are then used to produce a ratio. PIG is closely related to the LRF_adj_. Like the LRF_adj_, the PIG is unaffected by collinearity between treatment and surrogate [25].

## Meta-analytic approach: relative effect (RE, cf. R^2^_trial_) & adjusted association (AA, cf. R^2^_indiv_)

Buyse & Molenberghs (1998) [24] proposed two separate statistics in place of the PE: the relative effect (RE) and the adjusted association (AA) (Table 1). In contrast to Prentice, they reasoned that a useful surrogate should allow investigators to predict the treatment effect on a true end point, given knowledge only of the treatment and surrogate. This is the concept behind the RE, which is the ratio of the effect of treatment on true outcome over treatment on surrogate. An RE = 1 would indicate that the same magnitude of treatment effect operates on both the true outcome and surrogate end point, at a population, or ‘trial’ level. However, a value less than one would still allow prediction of treatment effects on the true outcome, provided the RE is estimated with little residual error. This may require large numbers of subjects. With this in mind, a similar statistic (R^2^_trial_) can be derived from multiple trials, or multilevel analysis of a single-large trial [27]. The RE relies on variability in the treatment effect on both surrogate and true end points. For an ideal trial level surrogate, α and β would have a monotonic relationship, with minimal residual error regardless of whether α and β are related linearly or nonlinearly [15].

Interestingly, the concept of an ideal trial level surrogate appears to be similar to that of nomic isomorphism. This is a special case of analogy, where source and target domains are interpretations of one physical theory. For example, volumetric flow and electric current are not only analogous concepts, they are also both described by the same mathematical equation [28].

Buyse and others also suggested that a useful surrogate should allow prediction of the true outcome for a particular individual, given knowledge of the treatment and surrogate. This is the idea behind the AA, which is the association between surrogate end point and true outcome, adjusted for the treatment. A perfect ‘individual level’ surrogate end point has a value for AA indicating no effect of treatment on the relationship between surrogate and true end points (∞ for binary end points, 1 for continuous end points) [24]. The corresponding meta-analytic statistic is R^2^_indiv_ [27]. As mentioned above, the AA is closely linked to the LRF [26]. Both RE and AA (R^2^_trial_ and R^2^_indiv_) can be applied to binary, ordinal and continuous end points [27].

**Figure 2:**
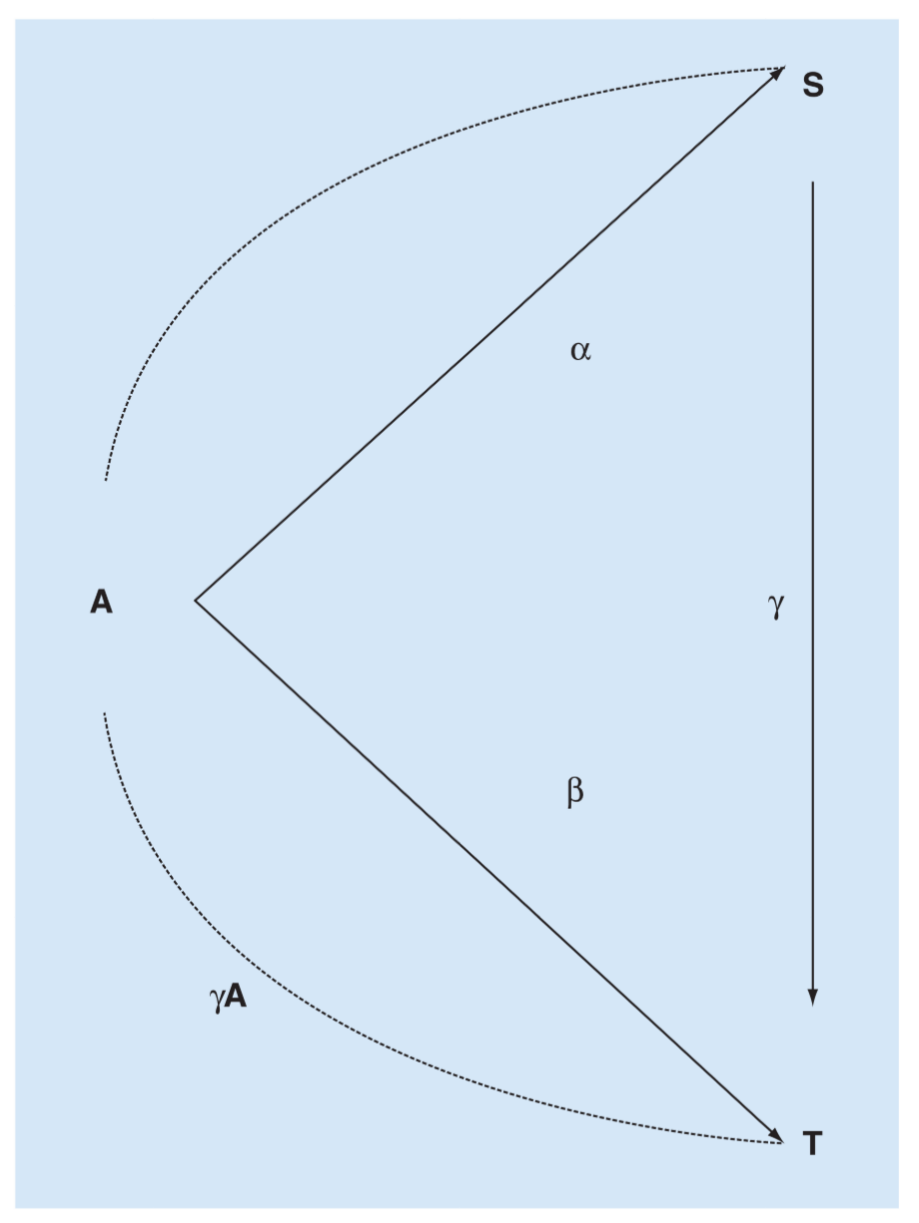
Modified directed acyclic graph showing relationships between treatment (A), surrogate (S) and true end point (T). A is assumed to influence T both directly, and also through S. The influence of unmeasured confounders is not included. α represents the association between A and S; β the association between A and T; and γ the association between S and T. β/α is the ratio of coefficients between A → S and A → T (the relative effect); γA is the association between S and T controlling for A (the adjusted association). Note that, in an RCT, the true end point (T) is usually the same as the clinical outcome (Z), and so there is only one triangle (connecting A, S and T) whereas in Figure 1, there are two (connecting A, S, T; and S, T and Z). Observational studies of associations between retina and brain allow the relationship between S and T to be evaluated in terms of both A and Z, while in an RCT the S → T relationship is only evaluated in terms of A. Redrawn from Figure 2 in [24].

Relationships envisioned by Buyse & Molenberghs (1998) [24] between treatment, surrogate and true end points can be illustrated (Figure 2).

The meta-analytic approach has the potential to give information about candidate surrogate end points that is more practical than hypothesis tests of Prentice’s criteria, or the PE. However in practice, evaluation of validity remains difficult [29], and although these statistics may give some useful information, they do not rule out the surrogate paradox [16].

## Qualitative evaluation of validity

Some authors recognize that it is not possible for a purely statistical definition of validity to fully evaluate whether or not a candidate surrogate end point is likely to be safe and effective [16,26]. At least two qualitative approaches have been proposed. These might be used instead of, or in addition to, attempts to measure statistical properties such as the LRF, PIG, AA or RE.

Wu *et al.* [30] described operational criteria to assess whether the direction of treatment effect on the surrogate is the same as that on the true end point. These are based on counterfactual terms, but can be assessed using procedures available in commercial statistical packages (e.g., generalized linear models).

**Figure 3:**
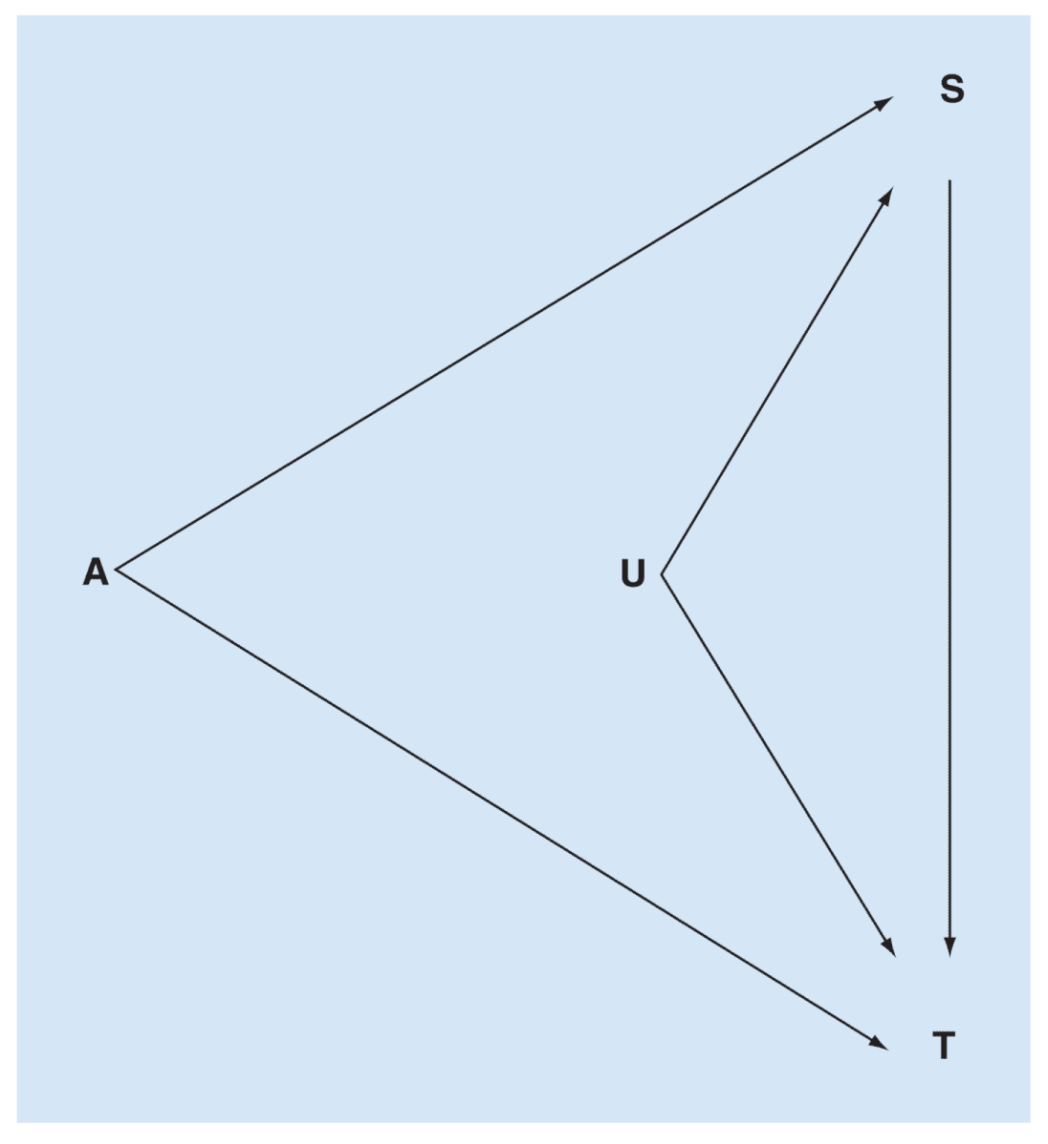
Directed acyclic graph showing relationships between treatment (A), surrogate (S) and true end point (T). Unmeasured confounders (U) of the relationship between S and T are included. Relationships between A and S, and A and T are assumed to be estimated under conditions of experimental randomization, and therefore not subject to confounding in the same way as S → T. The surrogate paradox can arise through: positive A → S → T effect, combined with a negative direct A → T effect; confounding of S → T by U; a lack of transitivity. In observational studies, A is not randomized and so the relationships A → S and A → T are also subject to unmeasured confounders. These should be described, as far as possible, in a structural model on the basis of *a priori* information about the biological context. Redrawn from Figure 2 in [16].

Vanderweele [16] discussed conditions that would be sufficient to ensure the surrogate paradox is avoided, and proposed three questions to assess the danger of being misled by a prospective surrogate (Figure 3):

- Might there be a negative direct effect of treatment on the true end point, not through the surrogate?
- Might the positive surrogate–true end point association be due to confounding?
- Might treatment affect the surrogate for different people than for whom the surrogate affects the true end point (i.e., a lack of transitivity)?

If the answer to all the three questions is ‘probably not’, one can have greater confidence that the surrogate end point does indeed reflect the true end point. This is a subjective assessment, based on *a priori* background information about relationships between treatment, surrogate, true end point and potential confounders.

For both Wu *et al.* [30] and VanderWeele [16], the conditions being assessed are sufficient, but not necessary. Failure to meet one (or more) does not prove a prospective surrogate is invalid. The criteria of Wu *et al.* [30] make use of data from previous RCTs, while Vander-Weele’s questions [16] rely on a *priori* knowledge of relationships between treatment, surrogate and true outcome.

This takes us back to considering the importance of the biological context, and the plausibility of analogy between what is measurable (e.g., the surrogate, the model animal), and what we would like to know with certainty but can only infer (e.g., the true end point, human biology). Like judgements about the validity of analogical inferences in general [28], when considering a surrogate end point the assessment of biological plausibility is ultimately subjective. The advantage of the questions proposed by VanderWeele is that they provide a systematic way to evaluate this context.

## Adaptation of surrogate methodology to evaluate retinal biomarkers of brain disease

### Strengths, weaknesses & theoretical pitfalls

Thinking of retinal variables as ‘surrogate-like’surrogate-like entities (or proxy markers) and analyzing them in terms of brain manifestation, disease exposure and outcome has several potential advantages.

By forcing researchers to explicitly describe the biological paradigm being assumed, this approach incorporates information about disease specific biological context into analyses of retina–brain associations. There is no guarantee that the assumed biological paradigm is correct. But discovering that a well-established retina–brain association does not hold when considered within the conventional biological context would be extremely useful, and could be investigated further by trying alternative frameworks that describe the data more accurately.

Showing that retinal variables are effectively equivalent to one or more brain variables (S⇔T), rather than just being directly associated, would allow investigators to reach much stronger conclusions about the meaning of retinal observations for a given disease. And, even if a retinal variable (S) did not contain a large amount of the information in the brain variable (T), with respect to exposure (A) and clinical outcome (Z), the assessment of these relationships would still provide a more detailed view of the links between retina and brain than that provided by simple univariate tests of S → T.

Having listed some strengths, several caveats should also be considered:

### Imperfect measurement of disease exposure

An RCT has an unambiguous treatment variable. It is clear who had treatment or placebo, and information about treatment assignment is fully captured by the treatment variable. In contrast, an observational study of disease mechanisms may measure several variables that are somehow related to disease severity, but neither any single variable nor the combination of all available variables may capture all the important information about the disease exposure acting on observed manifestations of the disease, or on the clinical outcome. In other words, definition of A is simple for a clinical trial but might be very complex for an observational study. This has implications for estimating coefficients between A and S, T or Z. A situation similar to the surrogate paradox could arise if A describes aspects of the disease that act to greater or lesser extent on S compared with T. For example, if A and S were positively associated, S and T were positively associated, but A and T were negatively associated (cf. VanderWeele’s first question).

This could be addressed through thorough research of the disease in question before deciding what variables to measure for A, S, T and Z. Investigators could consider using SEM to describe A as a latent variable, in order to summarize several observed variables that each describe part of a disease. They could also consider repeating the study in a separate validation cohort. In any case, publications arising from the analysis should clearly describe the rationale for choosing a particular variable to represent A, and explain the hazard of being misled, if this variable doesn’t fairly represent the disease in question with respect to S and T.

### Incorrect paradigm relating disease, retina, brain & outcome

VanderWeele’s first question [16] addresses the possibility of the surrogate paradox arising if there is a negative direct effect of A on T that is not mediated by S. In addition to imperfect disease exposure measurement, this situation could also occur if the theoretical framework relating A, S, T and Z did not account for real direct effects of A on Z.

This could be addressed by assessing alternative frameworks that include or exclude direct paths from A to Z.

### Lack of experimental randomization

In an RCT, the relationships A → S and A → T are estimated under experimental randomization, and the influence of confounders is controlled by design. Confounding could still occur for the relationship S → T, potentially resulting in the surrogate paradox (cf. VanderWeele’s second question). However, in an observational study, there is no experimental randomization and all relationships are susceptible to confounding. Statistical control cannot substitute for a lack of experimental control [15]. With this in mind, note that the approach of Wu *et al.* [30] involves inferences from pairwise measurements of association that can only be made under randomization, or by making untestable assumptions, and so is probably not suitable for observational data.

Again, this may be addressed by beginning with a thorough understanding of the biological context for the disease in question, and then designing a study to collect data on all known confounders of relationships between A, S, T and Z, so that these can be included in the analysis. Investigators could consider repeating the study in a separate validation cohort. The increased risk of bias and confounding inherent in observational studies compared with RCTs [31], should be clearly described, and specifically, that this particular analysis assumes there are no unmeasured confounders of relationships between A, S, T and Z.

### Lack of transitivity

The third route to the surrogate paradox [16] involves a situation where the relationship A → S is positive for different people than for whom S → T is positive (a lack of transitivity). This situation ought to be avoidable if paired data for A, S, T and Z are collected on subjects in an observational study.

## Proposed analytical framework

We now take pediatric cerebral malaria (CM) as a case study to illustrate how these concepts might be applied in practice.

### Biological context of CM

CM is a severe complication of infection with *Plasmodium falciparum*, characterized by coma, peripheral *P. falciparum* parasitemia, and absence of any other identified cause of coma [32]. It predominantly affects children in sub-Saharan Africa, and is an important contributor to the estimated 584,000 deaths worldwide from malaria in 2013 [33].

The mechanisms that allow the intravascular malaria parasite to cause coma and death remain unclear. However, sequestration of parasitized erythrocytes in the microvasculature of the CNS is accepted as the chief pathological feature of the disease [34]. It seems that, somehow, neurovascular sequestration causes injury to CNS parenchyma, which causes death. This constitutes a thumbnail sketch of a plausible biological paradigm for pediatric CM.

Measuring the severity of neurovascular sequestration in living patients is difficult. It may be possible to estimate total body parasite biomass by measuring a parasite-specific protein – HRP2 [35]. But, although HRP2 does seem able to predict and diagnose CM [36,37], plasma HRP2 cannot distinguish neurovascular sequestration from sequestration occurring in the rest of the body.

Several associations exist between retinal and brain manifestations of pediatric CM. For example, the presence of a characteristic malarial retinopathy accurately identifies true cases of pediatric CM from conditions with otherwise identical presentation [38], and the severity of retinal hemorrhages is directly correlated with the severity of cerebral hemorrhages at autopsy [39]. The severity of malarial retinopathy was recently shown to be directly associated with the severity of sequestration in both the retina and brain in fatal cases [40], suggesting that the retina may provide good biomarkers for cerebral sequestration and parenchymal injury in living patients. Furthermore, these associations are biologically plausible. They make sense in terms of anatomical and physiological similarities between discrete regions of retina and brain [6].

Having considered the biological context of malarial retinopathy in pediatric CM, how might we test retina–brain associations to evaluate the equivalence of retinal and cerebral tissue damage in this disease?

### Structure of disease paradigm & evaluation

Hypothetical relationships between disease severity (A), retina (S), brain (T) and outcome (Z) are illustrated in Figure 1. In this paradigm, both retina and brain are subject to proportional disease effects. Disease manifestations in retina and brain are directly associated, but cerebral disease manifestations alone cause death.

This framework can be evaluated in a number of ways, including simple tests of association between S and T that disregard other variables. Additional analyses could use surrogate-type statistics, such as the LRF or PIG, to estimate the degree to which S and T are interchangeable with regard to their relationship with A or Z. The ratio of coefficients for A → S and A → T could be calculated to derive a statistic similar in spirit to the RE. Importantly, both specific coefficients as well as the global fit of the whole paradigm can be evaluated using SEM, and the global fit can be compared with that of alternative frameworks (e.g., one including a direct A → Z path). The advantage of SEM is that it takes account of more complex (and realistic) relationships between variables than methods such as linear regression. Failure to include the correct confounding variables creates multivariate models that are prone to bias [41]. On the other hand, employing information about the biological context to specify a fuller model structure may provide a way to both limit bias and test the goodness of fit for the hypothetical biological paradigm.

## Conclusion & future perspective

Our approach of treating retinal variables as proxy markers of brain disease has the potential to describe relationships between prospective retinal biomarkers and brain disease features much more richly than simple tests of association, or even multiple regression models. Careful attention must be given to the biological context within which retina–brain associations are being tested, since inadequate variable measurement, and the impact of unmeasured confounders could lead to misleading results. However, this risk is arguably no greater than for conventional approaches. The risk may even be less, since the demand for an explicit description of the biological paradigm being hypothesized should at least help to clarify where assumptions might be less reliable.

Neurological biomarkers will be demanded with increasing urgency as industrialized populations continue to age, and growing numbers of people require investigations and treatments for neurodegenerative disease. So far, the retina has largely been investigated as a source of variables that are associated with brain dysfunction. If, as many authors seem to think, the retina truly is analogous to the brain, then we must develop methods to gain the clearest and most panoramic view through this biological window.

## Figures and Tables

**Table 1:** Several definitions of what it means for a biomarker to be a valid surrogate end point are listed. Each row describes one statistical approach, with operational criteria and comments. An explanation of statistical notation is given at the end of the table.

**Operational criteria or statistic**	**Comments**	**Ref.**
Prentice’s criteria (as expressed by f(T∣S) ≠ f(T) f(T∣S,Z) = f(T∣S)	A valid surrogate S should: Be associated with the true outcome T Completely capture the effect of treatment A on the true outcome T A candidate surrogate should be invalid if it fails to meet these criteria Potentially more useful for excluding poor surrogates (given enough power) than validating good ones The criteria (and related statistics) are not able to rule out the surrogate paradox [16]	[16]
PE PE = 1 – β_s_/β	A valid surrogate S should: Have a PE close to 1 Reflects the degree of treatment effect captured by the surrogate, rather than ruling out candidate surrogates that do not capture the entire treatment effect Can be applied to many types of data [24] The statistic is difficult to interpret, since it can lie outside 0–1, and will often have wide confidence intervals As well as describing the association between A and T given S, the PE also depends on the association between A and S. It operates best when there is little or no association between A and S [20,23,25]	[23]
LRF as interpreted by LRF = 1 – exp(-LRT(A,S:A)/n) LRF_adj_ = LRF/LRF_max_	A valid surrogate (at the ‘individual level’) should: Have an LRF close to 1 Reflects the degree of treatment effect captured by the surrogate, which is conceptually similar to the association between surrogate and true outcome, adjusted for treatment effect (both are expressed by f(T∣S,A)) The LRF reduces to the meta-analytic statistic R^2^_indiv_ for normally distributed end points [26] Can be applied to many types of data [26] As with the PE, a strong association between A and S will cause the LRF to approach 0. This is not the case for the LRF_adj_ in simulations [25]	[26] [25]
Proportion of information gain (PIG) PIG = LRT(*S* : 1)/LRT(*S,A* : 1)	A valid surrogate should: Have a PIG close to 1 A more straightforward way of expressing the LRF_adj_ [25]	[25]
RE AA RE = α/β AA = γA	A valid ‘trial level’ surrogate should: Have RE = 1, or any precisely estimated value Accurate estimation is likely to require data from multiple trials, or multilevel analysis of a single large trial. The meta-analytic statistic is R^2^_trial_ A valid ‘individual level’ surrogate should: Have AA = ∞ or 1 (depending on type of data) The AA is closely related to the LRF. The meta-analytic statistic is R^2^_indiv_ The RE and AA have the potential to give useful information in certain contexts. They cannot exclude the surrogate paradox [16]	[24]

Notation is defined as follows:

Prentice’s criteria

A: Treatment; S: Surrogate end point; T: True outcome.

f(T) signifies the probability distribution of T.

f(TIS) signifies the probability distribution of T, given S.

f(TIA) ≠ f(T) means the probability distribution of T given A is not equal to the probability distribution of T alone. That is, T is associated with Z.

f(T∣S,A) = f(T∣S) means the probability distribution of T given S and A is not different from the probability distribution of T given S. That is, A has no effect on T after adjustment for S.

Proportion of treatment effect (PE)

β_s_ is the regression coefficient of a model of A on T, adjusted for S.

β is the regression coefficient of a model of A on T unadjusted S.

Likelihood reduction factor (LRF)

LRT(A,S:A) is the likelihood ratio test statistic comparing a model of T given S and A, with a model of T given A

n = number of subjects.

LRF_max_ is the LRF of the best possible fitted model.

Proportion of information gain (PIG)

LRT(***S*** : 1) is the likelihood ratio test comparing a model of surrogate and intercept with a model including only the intercept.

LRT(***S,A*** : 1) is the likelihood ratio test comparing a model of surrogate, treatment and intercept with a model including only the intercept.

Relative effect, adjusted association (RE, AA)

β is the unadjusted estimate of the effect of A on T.

α is the unadjusted estimate of the effect of A on S.

β and α are estimated by separate logistic regression models.

γA is the effect of S on T adjusted for A, that is, f(T∣A,S).

## Executive summary

- The retina is often described as a ‘window to the brain’, implying that retinal features are analogous to equivalent brain features.
- Current research on retinal disease markers emphasizes direct associations between retinal and brain variables, without explicitly including information about biological context in the analysis.
- Proof of equivalency demands extra evidence, in addition to strong direct retina–brain associations.
- Thinking of retinal variables as proxy markers (or surrogate-like entities), that ought to provide a high proportion of information about corresponding brain variables, suggests that a range of existing statistical methods could find novel applications in the field of biomarker research.
- Our approach is illustrated, using pediatric cerebral malaria as an example.
